# Realizing nearly-zero dark current and ultrahigh signal-to-noise ratio perovskite X-ray detector and image array by dark-current-shunting strategy

**DOI:** 10.1038/s41467-023-36313-6

**Published:** 2023-02-06

**Authors:** Peng Jin, Yingjie Tang, Dingwei Li, Yan Wang, Peng Ran, Chuanyu Zhou, Ye Yuan, Wenjuan Zhu, Tianyu Liu, Kun Liang, Cuifang Kuang, Xu Liu, Bowen Zhu, Yang (Michael) Yang

**Affiliations:** 1 State Key Laboratory of Modern Optical Instrumentation, College of Optical Science and Engineering, Zhejiang University, Hangzhou, 310007 Zhejiang China; 2 Key Laboratory of 3D Micro/Nano Fabrication and Characterization of Zhejiang Province, School of Engineering, Westlake University, Hangzhou, 310024 Zhejiang China; 3 College of Information Science and Electronic Engineering, Zhejiang University, Hangzhou, 310007 Zhejiang China; 4 State Key Laboratory of Advanced Technology for Materials Synthesis and Processing, Wuhan University of Technology, Wuhan, 430070 PR China; 5 College of Electronic and Optical Engineering, and College of Flexible Electronics (Future Technology), Nanjing University of Posts and Telecommunications, Nanjing, 210023 P. R. China; 6 Intelligent Optics & Photonics Research Center Jiaxing Institute of Zhejiang University, Jiaxing, Zhejiang 314041 China

**Keywords:** Optoelectronic devices and components, X-rays, Imaging and sensing

## Abstract

Although perovskite X-ray detectors have revealed promising properties, their dark currents are usually hundreds of times larger than the practical requirements. Here, we report a detector architecture with a unique shunting electrode working as a blanking unit to suppress dark current, and it theoretically can be reduced to zero. We experimentally fabricate the dark-current-shunting X-ray detector, which exhibits a record-low dark current of 51.1 fA at 5 V mm^−1^, a detection limit of 7.84 nGy_air_ s^−1^, and a sensitivity of 1.3 × 10^4^ μC Gy_air_^−1^ cm^−2^. The signal-to-noise ratio of our polycrystalline perovskite-based detector is even outperforming many previously reported state-of-the-art single crystal-based X-ray detectors by serval orders of magnitude. Finally, the proof-of-concept X-ray imaging of a 64 × 64 pixels dark-current-shunting detector array is successfully demonstrated. This work provides a device strategy to fundamentally reduce dark current and enhance the signal-to-noise ratio of X-ray detectors and photodetectors in general.

## Abstract (web summary)

The high dark current of perovskite photodetectors hinders the full potential of perovskites as active material for X-ray detectors. Here, Jin et al. provide a strategy to reduce the dark current to zero and massively enhance the signal-to-noise ratio of perovskite X-ray detectors and photodetectors.

## Introduction

Metal halide perovskites (MHPs) have demonstrated highly desirable properties including large mobility-lifetime product, strong X-ray absorption, and easy synthesis, showing sensitive X-ray detection performance^1–21^. Compared with the most prevalent amorphous Selenium (*α-Se*) detectors, MHPs possess extraordinary sensitivity to X-ray and high stopping power to ionization radiation^22–25^. Low-temperature solution process fabrication enables MHPs the easy integration with Si-based application-specific integrated circuits (ASIC) for signal readout^17^. Currently, the most prevalent direct X-ray detection application is photoconductive-type flat-panel X-ray detectors^26,27^, they generally require dark current densities well below 1 nA cm^−2^ to maintain high detective quantum efficiency and dynamic range^28^. High sensitivity and low detection limit are also the key requirements to reduce the X-ray doses used on patients^3^. However, the high dark current of perovskite photoconduction detectors largely blocks their utilization in practical X-ray imagers^29^. First, a high dark current can quickly fill up the storage capacitance of TFT or CMOS pixels prior to X-ray illumination, which deteriorates the image contrast and dynamic range. Also, a large dark current significantly increases the shot noise and results in a poor signal-to-noise ratio (SNR)^30–32^. Lots of attention have been paid to reducing the dark current very recently, including 3D/2D perovskite heterojunction, dopant compensation of perovskite single crystals, and inserting insulating polymer between perovskite and electrode^33–39^. Given those encouraging progresses, the dark current density of perovskite X-ray detectors is still quite large, along with the notorious current drifting issue, becoming the biggest challenge toward its real-world applications.

**Fig. 1:**
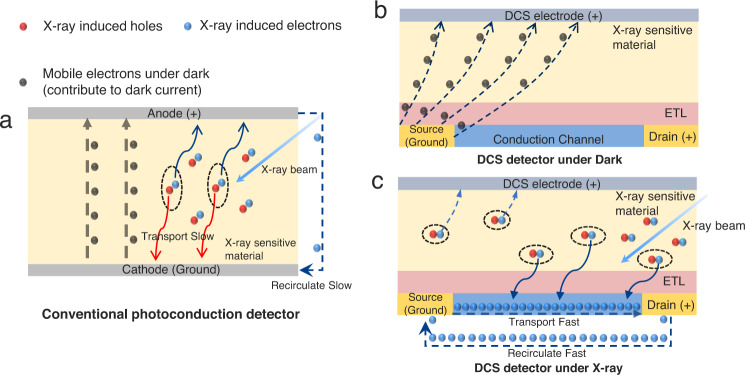
Working mechanism of dark-current-shunting (DCS) detector and conventional photoconduction detector. **a** Working mechanism of conventional photoconduction detector. The dark current and photocurrent are conducted in the same path and collected by the same electrodes. **b** Working mechanism of dark-current-shunting (DCS) detector. The electrons in the dark are emitted from the source and collected by the DCS electrode. **c** The X-ray induced electrons are generated from X-ray sensitive material and part of them drifted into a conduction channel with high carrier mobility under a built-in electric field between X-ray sensitive material and electron transport layer (ETL), then collected by drain electrode under an electric field applied in the lateral conduction channel. Even some of the X-ray induced electrons will be attracted to DCS electrode, with the high photoconductive gain effect of the conduction channel, the photocurrent is still strong.

In conventional photoconduction devices (Fig. 1a), the dark current and photocurrent are conducted in the same path and collected by the same electrodes, reducing the dark current is equivalent to increasing the resistance, which in turn also largely cuts the X-ray photocurrent. Herein, we propose a novel and general device structure, which can split the dark and photocurrent. It allows a fundamental and complete suppression of dark current (Fig. 1b). In this device structure, the electrons under dark are emitted from the source electrode and most of them can be collected by the top dark-current-shunting (DCS) electrode, or at least they will no longer be received by the drain, which gives the zero source-drain dark current. Under X-rays (Fig. 1c), the excited electrons, are first generated from X-ray-sensitive materials and transported to a lateral conduction channel with higher carrier mobility, thus, the captured carriers can transport rapidly in the conduction channel, reinject and recirculate swiftly between source and drain contacts, due to the long lifetime of the conduction channel, the captured carriers can transport longer before the recombination^40^. The amount of charges passing through the cross section of a conductor per unit of time is much greater. Therefore, these carriers in the conduction channel can offer much stronger photocurrent than the case with the mere X-ray sensitive materials^39–41^. Even some of the X-ray induced electrons will be attracted by DCS electrode, with the high photoconductive gain effect of the conduction channel, the photocurrent is still quite strong. Then the signal current is collected by the drain under an electric field applied from drain to source. In this scenario, the drain only receives X-ray-generated electrons, simultaneously gaining nearly-zero dark current and relatively high X-ray photocurrent.

In this study, we proposed and experimentally demonstrated the DCS detector that can effectively shunt the dark current, without much weakening of the photocurrent. The mobile electrons under dark can theoretically be fully depleted. The experimentally measured dark current is only 51.1 fA with no observable baseline drift, approaching the lowest measurable current of a typical semiconductor analyzer. As a result, the signal-to-noise ratio (SNR) was improved by two orders of magnitude. In addition to the high SNR single-pixel detector, we also successfully demonstrated a 64 × 64 image array with ~300 μm pixel pitch.

## Results and discussion

**Fig. 2:**
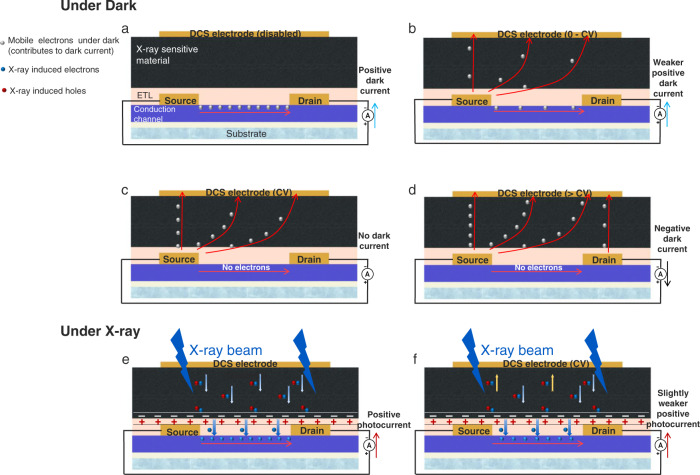
Detailed working principles of dark-current-shunting (DCS) X-ray detector. **a** Working principle at the dark condition and with DCS electrode disabled. The electrons emitted from the source transport through the conduction channel, and are collected by the drain, the DCS detector is working in a two-terminal mode similar as a photoconductive detector. **b** Working principle at the dark condition and with DCS electrode applied with a small bias. Some electrons emitted from the source are attracted by the DCS electrode, and the drain collects fewer electrons than the case described in Fig. 2a, leading to a smaller dark current. **c** Working principle at the dark condition and with DCS electrode applied with a CV. The electrons emitted from the source are all attracted by the DCS electrode, and the drain will not receive any electrons, leading to zero dark current in principle. **d** Working principle at the dark condition and with DCS electrode applied with a bias larger than CV. Some electrons are extracted from the drain and collected by the DCS electrode, leading to a negative dark current. **e** Working principle at X-ray illumination and with DCS electrode disabled. The DCS detector is working in a two-terminal mode similarly as the photoconduction detector. **f** Working principle at X-ray illumination and with DCS electrode applied with a CV. In order to minimize the dark current, the DCS electrode should be biased with a CV. Under X-ray excitation, the photo-generated excess carriers are transported through the transport layer (ETL) and sensitize the conduction channel, eventually producing photocurrent at the drain (Source electrode is always contacted to the ground, drain electrode is applied with a working voltage. Working voltage and CV here are positive).

At the dark condition, when the DCS electrode is disabled and a working voltage is applied to the drain, the electrons are emitted from the source, then transport through the conduction channel, and eventually collected by the drain (Fig. 2a), the measured drain current is positive. This is the typical working mechanism of a photoconductive detector with two lateral terminals. In our design, we add an extra DCS electrode on the top. By applying a small voltage to it, the electrons emitted from the source will be attracted by the DCS electrode. Because the mobile electrons under dark are shunted, there are fewer electrons being collected by the drain, thus the dark current in the actual conduction channel is suppressed (Fig. 2b). In principle, there is a critical voltage (CV), under which the mobile electrons under dark can be completely shunted by the top DCS electrode. In this circumstance, the drain will not receive any mobile electrons and the dark current can be zero (Fig. 2c). If the applied voltage on the DCS electrode increases further, the mobile electrons will be extracted from the drain to the DCS electrode (Fig. 2d), the dark current collected by the drain will become negative.

In order to minimize the absolute value of dark current and obtain a high SNR, the DCS electrode can be biased with a CV, making dark current fully shunted. Under X-ray, the excess carriers are generated in the photo-active material, those X-ray induced electrons are drifted when interfaced with the electron transport layer (ETL)^16^, and sensitize the lateral conduction channel, which means the photosensitive material generates an abundance of charges, these charges being captured by a transport layer with higher mobility can transport rapidly in the conduction and reinject and recirculate swift between metal contacts. Then, a photoconductive gain can be obtained. As a result, a strong X-ray photocurrent can be measured at the drain (Fig. 2e). It should be noted that the electric field applied by the DCS electrode will possibly attract some of the X-ray induced electrons and produce a lightly weaker photocurrent than the case with the DCS electrode disabled (Fig. 2f), but the overall SNR can still be significantly improved given the nearly completely suppressed dark current. The experimental evidence will be presented in the following section.

**Fig. 3:**
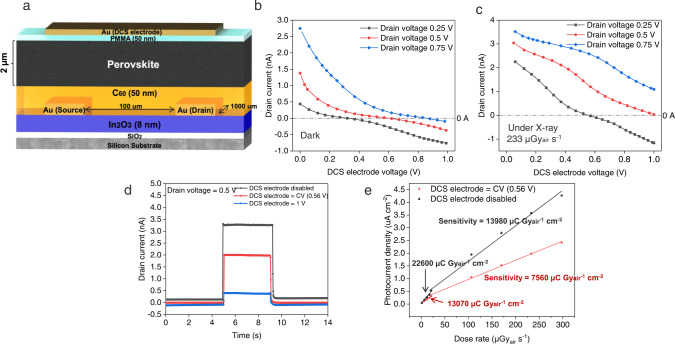
Schematic, current–voltage characteristics and sensitivity of DCS X-ray detectors. **a** Schematic of DCS X-ray detectors. **b** Current–voltage curves in terms of DCS electrode voltages and drain currents in dark conditions. The drain current decreases with increased DCS biases. **c** Current–voltage curves in terms of DCS electrode voltages and drain currents when exposed to X-ray. **d** X-ray generated pulse signals were illustrated when the DCS electrode was disabled, applied with a CV (0.56 V) and applied with a voltage larger than CV (1 V). The dose rate of X-ray was 233 μGy_air_ s^−1^. **e** The sensitivity of the DCS detectors was calculated when the DCS electrode was disabled and applied with a CV (0.56 V).

Following this design principle, we successfully fabricated DCS X-ray detectors with a structure as shown in Fig. 3a. Black-phase FA_0.92_Cs_0.04_MA_0.04_PbI_3_ perovskite was selected as an exemplary X-ray sensitive material in this study. Its X-ray diffraction (XRD) and photoluminescence (PL) are shown in Figs. S1 and 2. It has a density of ~4 g/cm^3^, providing a large linear attenuation coefficient of 10 cm^−1^ to 40 keV X-ray (Figure S3). In_2_O_3_ is a kind of semiconductor material which has been widely used as the channel material for thin-film transistors. And it merits of low-temperature solution-processability. The trapped carriers on its interface can alter the conductivity of the In_2_O_3_ channel through capacitive coupling and high photoconductive gain can be obtained^39^. The primary functional layers of this device, perovskite and In_2_O_3_ are both processed by solutions. The band diagram of the device in the equilibrium at dark and under X-ray^39^ are shown in Figure S4. The source electrode was always grounded, and the working voltage was applied to the drain during operation. As expected, first, the drain current decreased with the increased voltage applied to the DCS electrode (Fig. 3b). At a CV of ~0.56 V, the drain current was almost fully suppressed to be nearly-zero. With further elevated voltage at the DCS electrode, the drain current turned to be negative and its absolute value continuously increased. As shown in Fig. 3b, with varied drain-source voltages (0.25 V, 0.5 V, and 0.75 V), we can always find a critical DCS voltage (0.30 V, 0.56 V, and 0.85 V, respectively) to shunt the dark current completely. Those detectors are operating with the matched DCS and drain-source voltages under X-rays, and the photocurrent is always positive under this working condition as shown in Fig. 3c. Given a fixed working voltage (drain-source voltage) of 0.5 V, we tested the X-ray photocurrents with 0 V, 0.56 V, and 1 V bias voltages applied to the DCS electrode (Fig. 3d). It can be seen that when a CV was applied to the DCS electrode, the drain current was decreased to nearly-zero, and the X-ray photocurrent was only slightly diminished, compared with the case of the disabled DCS electrode. The sensitivity of our DCS X-ray detectors reaches 1.3 × 10^4^ μC Gy_air_^−1^ cm^−2^ under the circumstance that the dark current is decreased to be nearly-zero, which is very much different from the typical 2-terminal X-ray photoconduction detectors (Fig. 3e). As the X-ray dose rate is increased, the photocurrent demonstrates a sublinear dependence on it. This reduction in sensitivity can be explained in terms of trap states present either in In_2_O_3_ or at the interface between the In_2_O_3_ and the underlying SiO_2_ layer. Under high illumination intensities the density of available trap states is reduced, resulting in saturation of the photoresponse^41^. Although our device structure appears to be as similar as a top-gated phototransistor, its functioning mechanism is different since there is no gate regulation effect with this very thin PMMA spacing layer (Figure S5). Some of the devices may use the thick PMMA layer as a dielectric layer, but their PMMA layer is too thick (~250 nm) to allow the current pass through^40^. Our PMMA layer is only ~50 nm, it’s not insulative but may introduce a capacitance between DCS electrode and perovskite. The capacitance seems will not affect the final signal, which is collected in Drain electrode on the other side of the perovskite. Comparing the MHP transistor’s transfer curves^42^ to our device (Figure S5), there is no on/off state in our device when adjusting the DCS electrode’s voltage. The PMMA was mainly used as a buffer to avoid unexpected chemical corrosions between perovskite and metal. The device without PMMA also shows similar dark current shunting behaviors when a voltage is applied to the DCS electrode. Its dark current can also be suppressed to nearly-zero when the voltage of the DCS electrode reaches a critical value of ~0.4 V (Figure S6), given a working voltage of 0.5 V. In a conventional X-ray photoconductive detector, the dark current is basically determined by the effective resistance between two terminals. Although metal halide perovskites have shown many attractive characteristics as next-generation X-ray detectors, its resistivity is usually too small and is fluctuating because of the ion drift. Our strategy provides a unique solution to suppress the dark current from the perspective of device design, and the dark current in principle is no longer limited by the resistivity of detector materials.

**Fig. 4:**
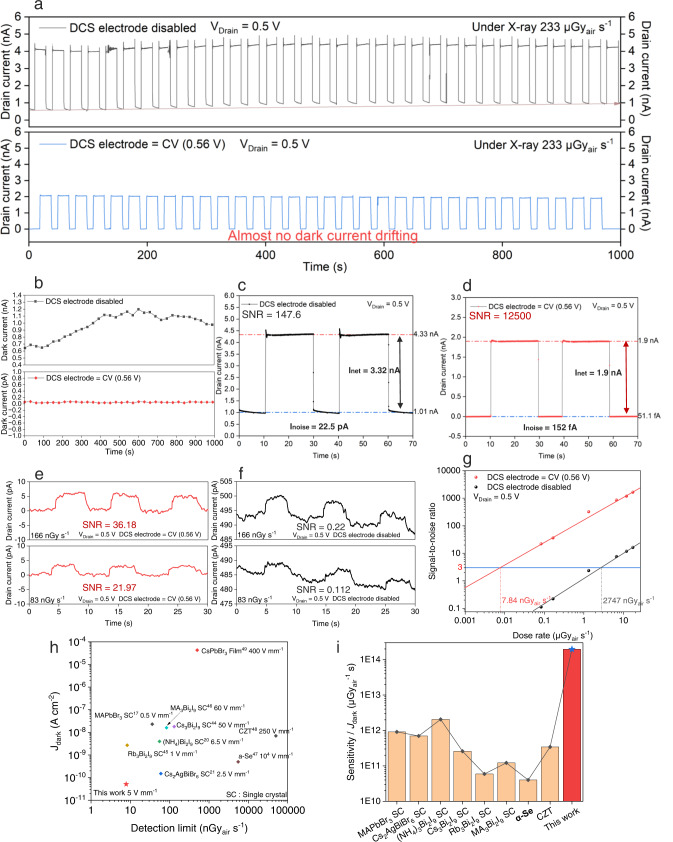
Characterizations of pulse-train response, signal-to-noise ratio (SNR), and lowest detection limit. **a** X-ray pulse-train response. By applying CV (0.56 V) to the DCS electrode, the signal-to-noise ratio (SNR), the pulse quality, and stability are obviously improved. There is almost no dark current drifting. **b** Characterization of dark current drifting. When the DCS electrode is disabled, the dark current gradually shifts from 0.615 nA to 0.986 nA. When the DCS electrode is biased with CV, the dark current reveals almost no shift during the pulse-train measurement. **c** The noise current and the SNR are 22.5 pA and 147.6, respectively, when the DCS electrode is disabled. **d** The noise current and the SNR are 0.152 pA and 12500, respectively, when DCS electrode is applied with a CV (0.56 V). **e** With the DCS electrode biased with a CV (0.56 V), the SNRs are 36.18 and 21.97 under respective X-ray dose rate of 166 nGy_air_ s^−1^ and 83 nGy_air_ s^−1^. **f** With the DCS electrode disabled, the SNRs are 0.22 and 0.112 under respective X-ray dose rate of 166 nGy_air_ s^−1^ and 83 nGy_air_ s^−1^. **g** Characterizations of detection limit. The detection limit (SNR = 3) of the DCS detector is as low as 7.84 nGy_air_ s^−1^, which is 350 times lower than the control photoconductor detector. The lowest detection limit was estimated by the reverse extension line to where the SNR = 3. **h** The comparison with respect to the dark current density and the detection limit between this work and other state-of-the-art photoconduction detectors is demonstrated. The applied electric fields are noted along with the detector materials. **i** The sensitivity/*J*_dark_, a figure of merit to evaluate SNR, is illustrated to compared our DCS detector and other reported state-of-the-art photoconduction detectors.

The dynamic response, SNR, lowest detection limit, and stability are evaluated in this secession. As shown in Fig. 4a, the device with a CV (0.56 V) applied to the DCS electrode clearly exhibits a much more stable signal current than the two-terminal photoconductor. The average photocurrent, dark current, and noise current are revealed. The net signal current (*I*_net_) was derived by subtracting the average photocurrent (${\bar{I}}_{{{{{{{\rm{photo}}}}}}}}$) by the average dark current (${\bar{I}}_{{{{{{{\rm{dark}}}}}}}}$).

1 $${I}_{{{{{{{\rm{net}}}}}}}}=\,{\bar{I}}_{{{{{{{\rm{photo}}}}}}}}-{\bar{I}}_{{{{{{{\rm{dark}}}}}}}}$$

The noise current (*I*_noise_) of was obtained by calculating the standard deviation of the dark current,

2 $${I}_{{{{{{{\rm{noise}}}}}}}}=\sqrt{\frac{1}{N}\mathop{\sum }\limits_{i}^{N}{\left({I}_{i}-{\bar{I}}_{{{{{{{\rm{dark}}}}}}}}\right)}^{2}}$$

The signal-to-noise ratio was calculated as:

3 $${{{{{{\rm{SNR}}}}}}}=\,{I}_{{{{{{{\rm{net}}}}}}}}/{I}_{{{{{{{\rm{noise}}}}}}}}$$

After applying CV to the DCS electrode, the dark current and noise current are as low as 51.1 fA and 152 fA (Figure S7), respectively, approaching the lowest measurable current of a typical semiconductor analyzer or source-meter. As a result, the SNR was improved by two orders of magnitude, showing an excellent signal output under the X-ray pulse train (Fig. 4a). When the DCS electrode is disabled, the dark current gradually shifts from 0.615 nA to 0.986 nA in the pulse-train measurement. While with the DCS electrode biased with the CV, the dark current reveals almost no shift (Fig. 4b). The noise current and the SNR of a single pulse photocurrent are 22.5 pA and 147.6, respectively, while the DCS electrode is disabled (Fig. 4c). But after the DCS electrode is biased with CV (0.56 V), its noise current and the SNR have tremendously improved to 0.152 pA and 12,500, respectively (Fig. 4d). The improved quality of signal current can also be verified under very low dose rate X-ray illumination. At the dose rate of 166 nGy_air_ s^−1^ and 83 nGy_air_ s^−1^, the respective SNRs of the DCS detector are 36.18 and 26.97, significantly larger than the SNRs of photoconductor detectors, which are 0.22 and 0.112, respectively, as shown in Fig. 4e and f. The detection limit of the DCS detector (SNR = 3) is as low as 7.84 nGy_air_ s^−1^ (Fig. 4g), which is 705 times lower than the standard dosage for X-ray medical examination^43^, and is 350 times lower than the control photoconductor detector. We compared our device with other reported state-of-the-art photoconductors in terms of dark current density, detection limit^17,20,21,44–49^ (Fig. 4h), as well as the sensitivity/*J*_dark_ (Fig. 4i) which is a figure of merit to evaluate the SNR. It is shown that our DCS detector delivers very low dark current, low detection limit, and ultrahigh SNR, even outperforming many previously reported state-of-the-art single crystal-based photoconductors. The DCS method is a universal device strategy and can be applied in other perovskite composition and conduction channel materials. We fabricated MAPbBr_3_, MAPbI_3_, and FA_0.92_Cs_0.04_MA_0.04_PbI_3_ on In_2_O_3_ and IGZO conduction channels, the electron transport layer is C_60_. The current–voltage curves in terms of DCS electrode voltages and drain currents of these devices were measured in the dark and under X-ray. It can be seen that the CV of these devices almost the same (0.35−0.4 V). All of the devices’ dark current can be suppressed to 0 A by applying DCS voltage (Figure S8). When the DCS electrode is biased with CV, all of the devices demonstrate stable current baselines and high signal-to-noise ratio (Figure S9). Although depositing high-quality thick perovskite films is quite challenging, and is not the main focus of this study, we preliminarily demonstrate the thick film-based detectors, those devices are configured with 80 µm or 200 µm MAPbI_3_/PMMA composite on top of SnO_2_ electron transporting layer and In_2_O_3_ channel layer. The thick perovskite was fabricated by blade-coating with MAPbI_3_ and PMMA polymer binding solution. When the DCS electrode is biased with CV, the dark current of thick film devices can also be suppressed to nearly zero and the device demonstrate stable current baselines and high signal-to-noise ratio. The CV seems not to be influenced by different compositions of materials, but will increase with larger conduction channel length, as well as thicker X-ray sensitive material (Figure S10), because stronger electric field are needed to attract the mobile electrons under dark to DCS electrode By using pure perovskite precursor solution, we varied the perovskite thicknesses between 300 nm to 2000 nm, we found that the thinner perovskite devices have lower sensitivity in lines with its different X-ray stopping powers (Figure S11), it implies that the built-in electric field between perovskite and C_60_ might strong enough to extract X-ray induced electrons close to the surface of 2 μm perovskite, we suggest the DCS detector may perform better by increasing perovskite thickness further while maintaining high film quality. To preliminarily investigate its spatial resolvability in scanning-based X-ray imaging, we measured its modulation transfer function (MTF) by scanning the object with a stepping motor (Figure S12a). The used line pair mask plate is shown in Figure S12b, the minimum resolved line pair is 5.5 lp mm^−1^ for a single-pixel device. We also compared our DCS detector with the typical vertically-stacked photoconductive detector with same perovskite composition and thickness (Figure S13a), whose current–voltage curves is shown in Figure S13b. Under the same 5 V mm^−1^ electrical field, the dark current density is 72 nA cm^−2^, that is more than a thousand time higher than our DCS device. Due to the high dark current and poor SNR, the control photoconductor device cannot generate neat signal output at the dose rate of 233 μGy_air_ s^−1^ (Figure S13c), and the dark current were shifting non-linearly from 1.05 nA to 1.23 nA during the pulse-train measurement, clearly, this sort of unpredictable baseline drift can’t be simply removed by applying an offset voltage in the external circuit.

**Fig. 5:**
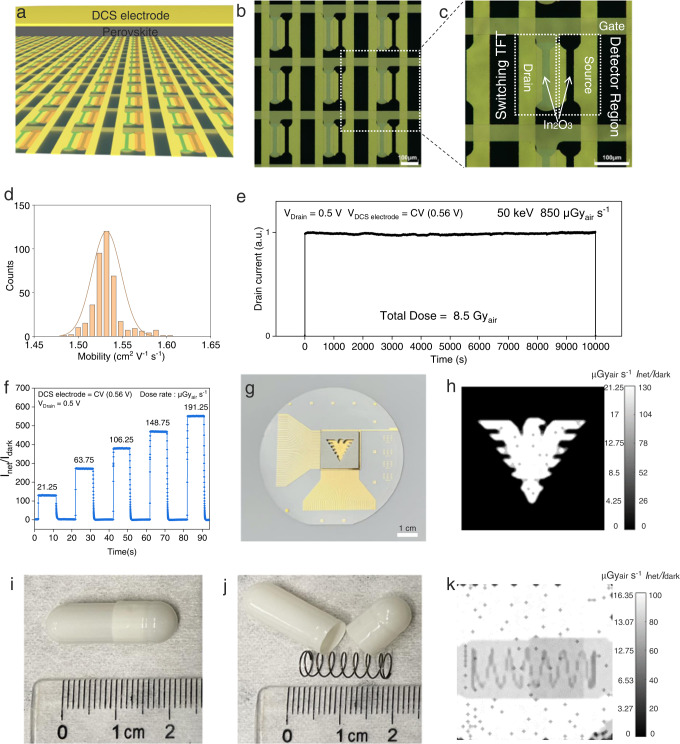
DCS X-ray detector array and X-ray imaging. **a** Schematic illustration of the DCS X-ray detector array. **b** Optical image of a fraction of detector array of nine pixels. **c**, The pixel pitch is 312.5 μm. The photosensitive area is 30 μm × 200 μm. **d** Electron mobility distribution of randomly selected 400 pixels. **e** Stability test of the DCS detector. The X-ray photon energy is 50 keV and the dose rate is 0.85 mGy_air_ s^−1^. The total received dose is 8.5 Gy_air_. **f** The image contrast is determined the value of *I*_net_/*I*_dark_ at different X-ray dose rates. **g** The optical image of the 64 × 64 detector array and a 2 mm thick stainless steel mask plate with a hollowed-out figure of ‘Qiushi Eagle’. **h** Obtained X-ray image while the DCS electrode is biased with CV (0.56 V). **i** Top view of the coil spring in the capsule. The coil spring cannot be seen by eyes. **j** Top view of the coil spring. **k** Obtained X-ray image of the coil spring in the capsule, with the DCS electrode biased with the CV (0.56 V).

Lastly, we fabricated a proof-of-concept 64 × 64 matrix DCS detector array in an area of 2 cm × 2 cm and obtained the X-ray imaging. A schematic illustration of the DCS X-ray detector array is demonstrated in Fig. 5a, the sectional view is shown in Figure S14, the pixel pitch is ~300 μm. The solution-processed 64 × 64 matrix In_2_O_3_ TFTs back panel with a pixel density of 1024 per square centimeter is shown in Figure S17a. The microscopic images of the X-ray detector array and a single-pixel are given in Fig. 5b and c. The transfer curves and the output curves of a single In_2_O_3_ transistor in the array were measured as shown in Figure S15. We also calculated the linear electron mobility (Fig. 5d), the subthreshold swing (Figure S16a), the threshold voltage (Figure S16b) and the current on/off ratio (Figure S16c) of 400 randomly picked pixels and illustrated the distributions of those parameters. 50 nm thick of C_60_ and 2 μm thick of perovskite were integrated with In_2_O_3_ TFTs. By using the Poly(perfluoroalkyl vinyl ether) (CYTOP) to cover the inactive region in the array, the perovskite precursor will not infiltrate into those areas and will only be deposited in the active region. This patterned-growth method contributes to a smooth and precisely covered perovskite layer (Figure S17b). Then a layer of Au (DCS electrode) was evaporated on top of the detector array (Figure S17c). The traditional a-Se array is fabricated by depositing a-Se on top of the a-Si TFT back panel, and then evaporating the top electrode as the common terminal of the photoconductor. Although our DCS single-pixel detector appears to have more complicated device structure, the fabrication of array detector is actually quite similar. We first fabricated the pixelized In_2_O_3_ TFT back panel, and then deposit the X-ray sensitive material (perovskite) onto the In_2_O_3_ TFT array and finally evaporate the top contact as the common DCS electrode. As for the readout circuit, the a-Se detector directly readout the charge (current) signal from drain electrode of the switching MOSFET, our device also readout the signal from drain electrode of the In_2_O_3_TFT. In addition, the analog data processing units including the preamplifier, Analog to Digital (AD) conversion can be shared with the conventional a-Si sensors. The preliminary stability test showed no observable degradations for a total received dose of 8.5 Gy_air_, the measurement was conducted in ambient air (Fig. 5e). The X-ray imaging contrast is determined by the value of *I*_net_/*I*_dark_ (Fig. 5f). A 2 mm thick stainless steel plate of ‘Qiushi Eagle’ (Figure S17d) was placed on top of the array and the image was captured at the dose rate of 21.25 μGy_air_ s^−1^ (Fig. 5g, h). The drain was applied a bias of 0.5 V, the DCS electrode was biased with 0.56 V, and the bottom gate of the switching TFT was biased with 20 V. The contrast ratio is tremendously improved and a clear image is acquired once the DCS electrode is applied with the CV. In order to demonstrate its capability of detecting an invisible object, we made a coil spring in the capsule, a commonly used demonstration in X-ray imaging test (Fig. 5i)^3,18^. The coil spring (Fig. 5j) is not observable by naked eyes but can be well visualized by our DCS image array under 21.25 μGy_air_ s^−1^ X-ray irradiation (Fig. 5k).

In conclusion, a novel device structure with a shunting electrode to split the flows of dark and photo currents is proposed and demonstrated in this study. It allows a tremendous suppression of the dark current to be nearly-zero with only slightly weakening of the photocurrent. A record low dark current of 51.1 fA (51.1 pA cm^−2^) and a noise current of 152 fA are experimentally demonstrated, the ratio of sensitivity/dark current representing the SNR of the device is outperforming previously reported photoconductive detectors by two orders of magnitude. In addition, our DCS detector shows no drift of baseline current that is often observed in typical perovskite photoconductive detectors. A DCS detector array with a pixel density of 1024 per square centimeter is successfully demonstrated with both solution-processed X-ray active material (perovskite) and electronic channel material (In_2_O_3_). This device strategy is not only a new perspective to solve the challenging issue in metal-halide X-ray detectors, but also should be generally effective in other types of photodetectors and material systems that require low dark current and high SNR.

## Methods

### Materials

Indium nitrate hydrate (In(NO_3_)_3_·xH_2_O, Aldrich, 99.999%) powder was dissolved in 2-methoxyethanol (2-ME) to prepare the In_2_O_3_ precursor solution (0.1 M). The IGZO precursor solution (0.1 M) was prepared by dissolving indium nitrate (In(NO_3_)_3_·xH_2_O, 99.999%), gallium nitrate (Ga(NO_3_)_3_·xH_2_O, 99.999%), and zinc acetate (Zn(CH_3_COO)_2_·2H_2_O, 99.999%) in 2-methoxyethanol (2-ME). The molar ratio of the In:Ga:Zn in the IGZO precursor solution was 9:1:2. The ITO precursor solution (0.1 M) was prepared by dissolving indium nitrate and stannous chloride (SnCl_2_·2H_2_O, 99.995%) in 2-methoxyethanol (2-ME). The molar ratio of the In:Sn in precursor solution was kept at 9:1. Then, acetylacetone and ammonium hydroxide were added as additives. Formamidinium iodide (FAI, >98.0%), Methylammonium iodide (MAI, >99.0%), Methylammonium bromine (MABr, >99.0%), Cesium iodide (CsI, >99.0%) and Lead(II) iodide (PbI_2_, 99.99%), Lead(II) bromine (PbBr_2_, 99.99%) were purchased from TCI. Fullerene-C_60_ (C_60_, 99.5%), Tin (IV) oxide (SnO_2_, 99.99%) was purchased from Sigma-Aldrich Company Ltd. Temozolomide (DMSO, 99%), N,N-Dimethylformamide (DMF, 98%) and chlorobenzene (CB, 99.8%) were purchased from J&K Scientific. Polymethyl methacrylate (PMMA, 495 A2 2%) was purchased from Nippon Kayaku Co. Ltd. Poly (perfluoroalkenyl vinyl ether) (CYTOP) was purchased from Asahi glass company (Japan) and consists of CTL-809M (solute) and CT-Solv.180 (solvent).

### Single-pixel device fabrication

The X-ray detectors were fabricated with a bottom gate top contact (BGTC) structure. 100 nm SiO_2_ was deposited on silicon (P^++^) substrate by PECVD at 350 °C as a dielectric layer. After O_2_ plasma treatment for 3 min, the In_2_O_3_, IGZO, ITO precursor solution filtered through a 0.2-μm syringe filter was spin-coated at 3000 rpm for 30 s on the substrate as a channel layer. After spin-coating, the In_2_O_3_, IGZO, ITO thin film was annealed at 100 and 200 °C for 1 min and 1 h, respectively. And the channel layer was patterned via a conventional photolithography process (AZ-1518 photoresist, 90 °C baking, AZ 300MIF developer) and etched in a mixed solution of hydrochloric acid and deionized water (1: 10, v-v) for 10 s. Next, Ni (3 nm) and Au (50 nm) were sequentially deposited as source/drain by electron-beam evaporation and patterned by lift-off photolithography (AR-P 5350, 105 °C baking, AR 300-26 developer), forming channel W/L of 1000/100 μm. Afterward, 50 nm of C_60_ was thermally evaporated in a separate vacuum chamber (<5 × 10^−4^ Pa) in sequence. The SnO_2_ solution was spin-coated by one step program at 4000 rpm for 30 s, and then annealed at 150 °C for 30 min. The FA_0.92_Cs_0.04_MA_0.04_PbI_3_ perovskite precursor (2.8 M) solution prepared by mixing CsI (29.1 mg) MAI (17.8 mg) FAI (443 mg) PbI_2_ (1290.83 mg) in 600 μL DMF and 400 μL DMSO, the FA_0.92_Cs_0.04_MA_0.04_PbI_3_ perovskite precursor (2.0 M) solution prepared by mixing CsI (29.1 mg) MAI (17.8 mg) FAI (443 mg) PbI_2_ (1290.83 mg) in 1120 μL DMF and 280 μL DMSO, the FA_0.92_Cs_0.04_MA_0.04_PbI_3_ perovskite precursor (1.4 M) solution prepared by mixing CsI (29.1 mg) MAI (17.8 mg) FAI (443 mg) PbI_2_ (1290.83 mg) in 1600 μL DMF and 400 μL DMSO, the MAPbI_3_ perovskite precursor (2.0 M) solution prepared by mixing MAI (317.94 mg) PbI_2_ (922.02 mg) in 800 μL DMF and 200 μL DMSO, the MAPbBr_3_ perovskite precursor (2.0 M) solution prepared by mixing MABr (223.94 mg) PbBr_2_ (734.02 mg) in 800 μL DMF and 200 μL DMSO. The precursors were stirred at room temperature for 120 min and filtered with a 0.22 μm PTFE filter prior to use. 500 nm, 1 and 2 μm FA_0.92_Cs_0.04_MA_0.04_PbI_3_: 35 μL FA_0.92_Cs_0.04_MA_0.04_PbI_3_ precursor solution (2.8 M for 2 μm and 2.0 M for 1 μm, 1.4 M for 500 nm) was spin-coated on top of the C_60_ layer by a two-consecutive step program at 1000 rpm for 10 s and 4000 rpm for 40 s (1.4 M for 30 s), the antisolvent CB was added at a 5-s countdown. The devices were immediately annealed on a hotplate at 100 ^◦^C for 10 min. 1 μm MAPbI_3_: 35 μL MAPbI_3_ precursor solution was spin-coated on top of the C_60_ layer by one step program at 4000 rpm for 30 s, the antisolvent CB was added at a 15 s countdown. The devices were immediately annealed on a hotplate at 100 ^◦^C for 10 min. 1 μm MAPbBr_3_: 35 μL MAPbBr_3_ precursor solution was spin-coated on top of the C_60_ layer by one step program at 4000 rpm for 30 s, the antisolvent CB was added at a 15 s countdown. The devices were immediately annealed on a hotplate at 100 ^◦^C for 10 min. 80 μm and 200 μm MAPbI_3_: MAI (477 mg) and PbI2 (1393 mg) dissolved in 3 mL GBL and stirred at room temperature for 120 min and filtered with a 0.22 μm PTFE filter. 300 μL solution was added to 3 mL Toluene in centrifuge tube, then centrifuged at 8000 rpm for 1 h. The liquid part was replaced by Toluene and then centrifuged at 8000 rpm for 1 h again. Afterward, the MAPbI_3_ powder was taken out and dried out and then added into the PMMA and Toluene mixing solution and stirred at room temperature for 2 h. Then the MAPbI_3_ precursor solution was blade coated on SnO_2_/In_2_O_3_ TFT substrate. The distance of the blade from the substrate were 500 μm (film dried out was 80 μm) and 900 μm (film dried out was 200 μm) and then dried out in the air for a day. 50 nm of PMMA spin-coated by 4000 rpm for 40 s was coated above, then a 100 nm Au has evaporated above with a mask plate covered with the area of the drain, source and the conduction channel.

### Detector array fabrication

The glass substrate (3 inch) was cleaned by immersing into acetone, isopropanol, and deionized water for 10 min, respectively, with ultrasonication. First, the back gate electrode region is patterned by using the standard lithography process. Next, the Ni/Au (5/30 nm) gate electrodes were evaporated by electron beam (Ei-5z, ULVAC), and then lifted off. Afterward, 100 nm SiO_2_ gate insulator was deposited by plasma-enhanced chemical vapor deposition (PECVD; SAMCO Inc. PD-220NL) at 300 °C. And the prepared In_2_O_3_ precursor solution was spin-coated as the semiconductor layer at 3000 rpm for 30 s and pre-baked on a hotplate at 200 °C for 5 min. The photoresist (AZ-1518) etch mask layer was spin-coated on the In_2_O_3_ thin film (4000 rpm, 60 s) and patterned by a mask aligner (Karl Suss MA6 mask aligner). Subsequently, the In_2_O_3_ thin film at the unwanted area was removed by the mixed solution of hydrochloric acid and deionized water (HCl:H_2_O = 1:10, v-v) for 10 s. And the photoresist was removed by rinsing with acetone, isopropyl alcohol, and deionized water in a sequential way. Then the patterned In_2_O_3_ thin film were annealed at 300 °C for 1 h. The Ni/Au drain/source electrodes (5/50 nm) were deposited and patterned, using the same process used for bottom gate electrodes, forming channel W/L of 150/20 µm. Finally, the SU-8 2000.5 photoresist (500 nm) was spin-coated and patterned by photolithography to protect the transistor. For patterning the perovskite area, the CYTOP was spin-coated onto the device and patterned by O_2_ plasma (PC-300, SAMCO Inc.). Subsequently, 50 nm C_60_ as electron transport layer was deposited by thermal evaporation with the patterned shadow mask in a separate vacuum chamber (<5 × 10^−4^ Pa). Afterward, the perovskite precursor solution prepared by mixing CsI (29.1 mg) MAI (17.8 mg) FAI (443 mg) PbI_2_ (1290.83 mg) in 600 μL DMF and 400 μL DMSO was stirred at room temperature for 120 min and filtered with a 0.22 μm PTFE filter prior to use. 60 μL precursor solution was spin-coated on top of the C_60_ layer by a two-consecutive step program at 1000 rpm for 10 s and 4000 rpm for 40 s, the antisolvent CB was added at a 5-s countdown. The devices were immediately annealed on a hotplate at 100 ^◦^C for 10 min. 50 nm of PMMA spin-coated by 4000 rpm for 40 s was coated above, then Ni/Au (5/50 nm) has evaporated above with a mask plate covered with the area of the drain, source, and the conduction channel.

### X-ray photocurrent measurement

The X-ray source was used as a commercially available Amptek Mini-X tube with an Ag target and 4 W maximum power output. The total X-ray dose was modulated by changing the current of the X-ray tube (5−80 μA), as well as the distance between the device and the X-ray source. The radiation dose rate was calibrated by using a radical ion chamber dosimeter. In the stability test, the energy of the used X-rays is 50 keV. On the other measurement, the energy of the used X-rays is 40 keV. The used output spectrum at different voltages is shown in Figure S18. The transfer curves, output curves, the voltage and current of drain and DCS electrode of the devices were regulated and measured by Keithley 2636B sourcemeter. For realizing data collection of the 64 × 64 active-matrix array, a data acquisition system was constructed by three PXI source measure unit (NI PXIe-4138) and a PXI matrix switch module (NI PXIe-2531) assembled in a PXI Chassis (NI PXIe-1073). Flexible cables were connected to the source, drain, and DCS electrode of the multiplexed detector using anisotropic conducting paste. The images detected by the detectors array were processed and median filtered by Matlab R2020a.

### Characterization of the materials

The FA_0.92_Cs_0.04_MA_0.04_PbI_3_ films were made on ITO substrates. The pre-patterned ITO substrates (1.5 × 1.5 cm^2^) were bought from PsiOTec Ltd and ultrasonically cleaned with soap, deionized water, acetone, and IPA in succession for 10 min. The as-cleaned ITO substrates were treated with UV-O_3_ for 30 min and transferred to a N_2_-filled glovebox. The XRD measurements of FA_0.92_Cs_0.04_MA_0.04_PbI_3_ were performed with Rigaku D/Max-B X-ray diffractometer with Bragg–Brentano parafocusing geometry, a diffracted beam monochromator, and a conventional cobalt target X-ray tube set to 40 kV and 30 mA. The photoluminescence (PL) was detected by Ocean Optics QE Pro. The 540 nm excitation light was made by Pico Quant PDL 800-D.

## Supplementary information

Supplementary Information

Peer Review File

## Source data

Source Data

## Data Availability

All data generated or analyzed during this study are included in this published article (and its supplementary information file). Source data are provided with this paper.

## References

[1] Zheng, X. et al. Ultrasensitive and stable X-ray detection using zero-dimensional lead-free perovskites. *J. Energy Chem.* **49**, 299–306 (2020).

[2] Yang, B. et al. Heteroepitaxial passivation of Cs_2_AgBiBr_6_ wafers with suppressed ionic migration for X-ray imaging. *Nat. Commun.* **10**, 1989 (2019). 31040278 10.1038/s41467-019-09968-3 PMC6491557

[3] Wei, H. T. & Huang, J. S. Halide lead perovskites for ionizing radiation detection. *Nat. Commun.* **10**, 12 (2019). 30842411 10.1038/s41467-019-08981-w PMC6403296

[4] Wei, H. T. et al. Sensitive X-ray detectors made of methylammonium lead tribromide perovskite single crystals. *Nat. Photon.* **10**, 333–339 (2016).

[5] Tie, S. et al. Robust fabrication of hybrid lead-free perovskite pellets for stable X-ray detectors with low detection limit. *Adv. Mater.* **32**, e2001981 (2020). 32588518 10.1002/adma.202001981

[6] Tie, S. et al. Efficient X-ray attenuation lead-free AgBi_2_I_7_ halide rudorffite alternative for sensitive and stable X-ray detection. *J. Phys. Chem. Lett.* **11**, 7939–7945 (2020). 32842746 10.1021/acs.jpclett.0c02343

[7] Heo, J. H. et al. High-performance next-generation perovskite nanocrystal scintillator for nondestructive X-ray imaging. *Adv. Mater.* **30**, 1801743 (2018). 10.1002/adma.201801743 30141200

[8] Song, X. et al. Metal-free halide perovskite single crystals with very long charge lifetimes for efficient X-ray imaging. *Adv. Mater.* **32**, e2003353 (2020). 32930461 10.1002/adma.202003353

[9] Pan, W. et al. Hot-pressed CsPbBr_3_ quasi-monocrystalline film for sensitive direct X-ray detection. *Adv. Mater.* **31**, e1904405 (2019). 31523875 10.1002/adma.201904405

[10] Tsai et al. A sensitive and robust thin-film x-ray detector using 2D layered perovskite diodes. *Sci. Adv.* **6**, eaay0815 (2020). 32300647 10.1126/sciadv.aay0815 PMC7148088

[11] Liu, J. Y. et al. Flexible, printable soft-X-ray detectors based on all-inorganic perovskite quantum dots. *Adv. Mater.* **31**, 8 (2019). 10.1002/adma.201901644 31169936

[12] Li, X. et al. All‐perovskite integrated X‐ray detector with ultrahigh sensitivity. *Adv. Opt. Mater.* **8**, 2000273 (2020).

[13] Hu, M. et al. Large and dense organic-inorganic hybrid perovskite CH_3_NH_3_PbI_3_ wafer fabricated by one-step reactive direct wafer production with high X-ray sensitivity. *ACS Appl. Mater. Interfaces* **12**, 16592–16600 (2020). 32216332 10.1021/acsami.9b23158

[14] Wei, H. et al. Sensitive X-ray detectors made of methylammonium lead tribromide perovskite single crystals. *Nat. Photon.* **10**, 333–339 (2016).

[15] Shrestha, S. et al. High-performance direct conversion X-ray detectors based on sintered hybrid lead triiodide perovskite wafers. *Nat. Photon.* **11**, 436–440 (2017).

[16] Zhao, J. et al. Perovskite-filled membranes for flexible and large-area direct-conversion X-ray detector arrays. *Nat. Photon.* **14**, 612–617 (2020).

[17] Wei, W. et al. Monolithic integration of hybrid perovskite single crystals with heterogenous substrate for highly sensitive X-ray imaging. *Nat. Photon.* **11**, 315–321 (2017).

[18] Peng, J. et al. Crystallization of CsPbBr_3_ single crystals in water for X-ray detection. *Nat. Commun.* **12**, 1531 (2021). 33750768 10.1038/s41467-021-21805-0 PMC7943776

[19] Deumel, S. et al. High-sensitivity high-resolution X-ray imaging with soft-sintered metal halide perovskites. *Nat. Electron.* **4**, 681–688 (2021).

[20] Zhuang, R. et al. Highly sensitive X-ray detector made of layered perovskite-like (NH_4_)_3_Bi_2_I_9_ single crystal with anisotropic response. *Nat. Photon.* **13**, 602–608 (2019).

[21] Pan, W. et al. Cs_2_AgBiBr_6_ single-crystal X-ray detectors with a low detection limit. *Nat. Photon.* **11**, 726–732 (2017).

[22] Basiricò, L. et al. Direct X-ray photoconversion in flexible organic thin film devices operated below 1 V. *Nat. Commun.* **7**, 13063 (2016). 27708274 10.1038/ncomms13063 PMC5059709

[23] Hunter, D. M., Belev, G., Kasap, S. & Yaffe, M. J. Measured and calculated K-fluorescence effects on the MTF of an amorphous-selenium based CCD x-ray detector. *Med. Phys.* **39**, 608–622 (2012). 22320770 10.1118/1.3673957

[24] Que, W. & Rowlands, J. X‐ray imaging using amorphous selenium: inherent spatial resolution. *Med. Phys.* **22**, 365–374 (1995). 7609716 10.1118/1.597471

[25] Donovan, J. L. Xeroradiographic properties of amorphous selenium. *J. Appl. Phys.* **41**, 2109–2114 (1970).

[26] Antonuk, L. E. Electronic portal imaging devices: a review and historical perspective of contemporary technologies and research. *Phys. Med. Biol.* **47**, R31–R65 (2002). 11936185

[27] Ou, X. et al. High-resolution X-ray luminescence extension imaging. *Nature* **590**, 410–415 (2021). 33597760 10.1038/s41586-021-03251-6

[28] Kasap, S. et al. Amorphous and polycrystalline photoconductors for direct conversion flat panel X-ray image sensors. *Sensors* **11**, 5112–5157 (2011). 22163893 10.3390/s110505112 PMC3231396

[29] Wu, H. D., Ge, Y. S., Niu, G. D. & Tang, J. Metal halide perovskites for X-ray detection and imaging. *Matter* **4**, 144–163 (2021).

[30] Fang, Y. & Huang, J. Resolving weak light of sub-picowatt per square centimeter by hybrid perovskite photodetectors enabled by noise reduction. *Adv. Mater.* **27**, 2804–2810 (2015). 25786908 10.1002/adma.201500099

[31] Fang, Y. J., Armin, A., Meredith, P. & Huang, J. S. Accurate characterization of next-generation thin-film photodetectors. *Nat. Photon.* **13**, 1–4 (2019).

[32] Thompson, M., Ellison, S. L. R. & Wood, R. Harmonized guidelines for single-laboratory validation of methods of analysis (IUPAC Technical Report). *Pure Appl. Chem.* **74**, 835–855 (2002).

[33] Li, Y. L. et al. Direct conversion X-ray detectors with 70 pA cm^−2^ dark currents coated from an alcohol-based perovskite ink. *J. Mater. Chem. C.* **10**, 1228–1235 (2022).

[34] Zhou, Y. et al. Heterojunction structures for reduced noise in large-area and sensitive perovskite x-ray detectors. *Sci. Adv.* **7**, eabg6716 (2021). 34516903 10.1126/sciadv.abg6716 PMC8442865

[35] Wei, H. T. et al. Dopant compensation in alloyed CH_3_NH_3_PbBr_3_-xClx perovskite single crystals for gamma-ray spectroscopy. *Nat. Mater.* **16**, 826–833 (2017). 28671663 10.1038/nmat4927

[36] Kim, Y. C. et al. Printable organometallic perovskite enables large-area, low-dose X-ray imaging. *Nature* **550**, 87–91 (2017). 28980632 10.1038/nature24032

[37] Zheng, X. P. et al. Defect passivation in hybrid perovskite solar cells using quaternary ammonium halide anions and cations. *Nat. Energy* **2**, 17102 (2017).

[38] Xu, Q. et al. CsPbBr_3_ single crystal X-ray detector with Schottky Barrier for X-ray imaging application. *ACS Appl. Electron. Mater.* **2**, 879–884 (2020).

[39] Peng, J. et al. Solution-processed perovskite/metal-oxide hybrid X-ray detector and array with decoupled electronic and ionic transport pathways. *Small Methods* **6**, 2200500 (2022). 10.1002/smtd.202200500 35754169

[40] Gao, Y. et al. Ultrathin and ultrasensitive direct X-ray detector based on heterojunction phototransistors. *Adv. Mater.* **33**, 2101717 (2021). 10.1002/adma.202101717 34219296

[41] Lopez-Sanchez, O. et al. Ultrasensitive photodetectors based on monolayer MoS_2_. *Nat. Nanotech.* **8**, 497–501 (2013). 10.1038/nnano.2013.100 23748194

[42] Liu, A. et al. High-performance inorganic metal halide perovskite transistors. *Nat. Electron.* **5**, 78–83 (2022).

[43] Wall, B. F. & Hart, D. Revised radiation doses for typical X-ray examinations. Report on a recent review of doses to patients from medical X-ray examinations in the UK by NRPB. National Radiological Protection Board. *Br. J. Radiol.* **70**, 437–439 (1997). 9227222 10.1259/bjr.70.833.9227222

[44] Zhang, Y. et al. Nucleation-controlled growth of superior lead-free perovskite Cs_3_Bi_2_I_9_ single-crystals for high-performance X-ray detection. *Nat. Commun.* **11**, 2304 (2020). 32385231 10.1038/s41467-020-16034-w PMC7210296

[45] Liu, Y. et al. Inch-size 0D-structured lead-free perovskite single crystals for highly sensitive stable X-ray imaging. *Matter* **3**, 180–196 (2020).

[46] Xia, M. et al. Unveiling the structural descriptor of A_3_B_2_X_9_ perovskite derivatives toward X-ray detectors with low detection limit and high stability. *Adv. Funct. Mater.* **30**, 1910648 (2020).

[47] Kasap, S. et al. Amorphous selenium and its alloys from early xeroradiography to high resolution X-ray image detectors and ultrasensitive imaging tubes. *Phys. Status Solidi B* **246**, 1794–1805 (2009).

[48] Tokuda, S., Kishihara, H., Adachi, S. & Sato, T. Preparation and characterization of polycrystalline CdZnTe films for large-area, high-sensitivity X-ray detectors. *J. Mater. Sci.-Mater. El* **15**, 1–8 (2004).

[49] Matt, G. J. et al. Sensitive direct converting X-ray detectors utilizing crystalline CsPbBr3 perovskite films fabricated via scalable melt processing. *Adv. Mater. Interfaces* **7**, 1901575 (2020).

