# Supplementary material for: Realizing nearly-zero dark current and ultrahigh signal-to-noise ratio perovskite X-ray detector and image array by dark-current-shunting strategy
Source: Nat Commun. 2023 Feb 6;14:626. doi: 10.1038/s41467-023-36313-6 (PMC9902443; doi:10.1038/s41467-023-36313-6)
Supplement: Supplementary file 1 — Supplementary Information [file 41467_2023_36313_MOESM1_ESM.pdf]

Supplementary Information

Realizing Nearly-Zero Dark Current and Ultrahigh Signal-to-Noise Ratio Perovskite X-ray Detector and Image Array by Dark-Current-Shunting Strategy

Peng Jin[1†], Yingjie Tang[2,3†], Dingwei Li[2,3], Yan Wang[2,3], Peng Ran[1], Chuanyu Zhou[1], Ye Yuan[4], Wenjuan Zhu[1,5], Tianyu Liu[1], Kun Liang[2,3], Cuifang Kuang[1,6], Xu Liu[1], Bowen Zhu[2]* & Yang (Michael) Yang[1,6]*

†Those authors contributed equally to this work.

[1]State Key Laboratory of Modern Optical Instrumentation, College of Optical Science and Engineering, Zhejiang University, Hangzhou 310007, Zhejiang, China.

[2]Key Laboratory of 3D Micro/Nano Fabrication and Characterization of Zhejiang Province, School of Engineering, Westlake University, Hangzhou 310024, Zhejiang, China.

[3]College of Information Science and Electronic Engineering, Zhejiang University, Hangzhou 310007, Zhejiang, China.

[4]State Key Laboratory of Advanced Technology for Materials Synthesis and Processing, Wuhan University of Technology, Wuhan 430070, PR China

[5]College of Electronic and Optical Engineering, and College of Flexible Electronics (Future Technology), Nanjing University of Posts and Telecommunications, Nanjing 210023, P. R. China

[6]Intelligent Optics & Photonics Research Center Jiaxing Institute of Zhejiang University, Jiaxing, Zhejiang, 314041 China

Corresponding Author:

Email:yangyang15@zju.edu.cn[1]

Email:zhubowen@westlake.edu.cn[2]

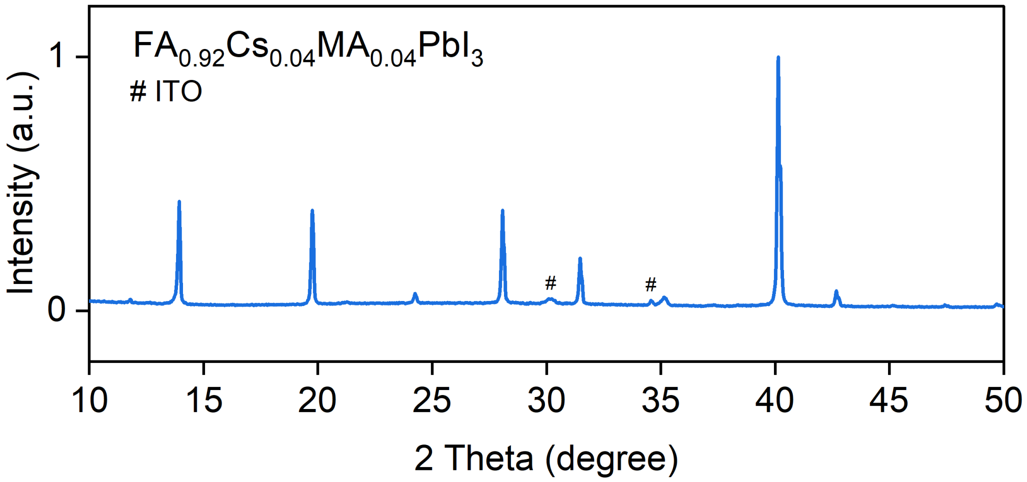

Figure S1. XRD of $FA_{0.92}Cs_{0.04}MA_{0.04}PbI_3$.

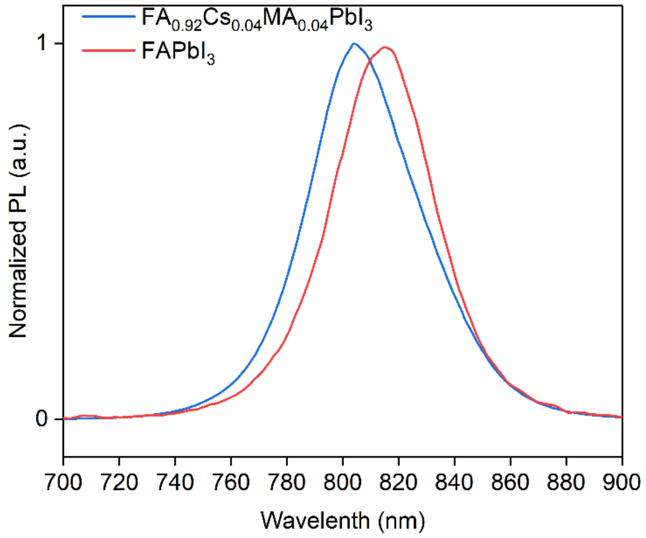

Figure S2. Photoluminescence (PL) of $FA_{0.92}Cs_{0.04}MA_{0.04}PbI_3$ and pure $FAPbI_3$.

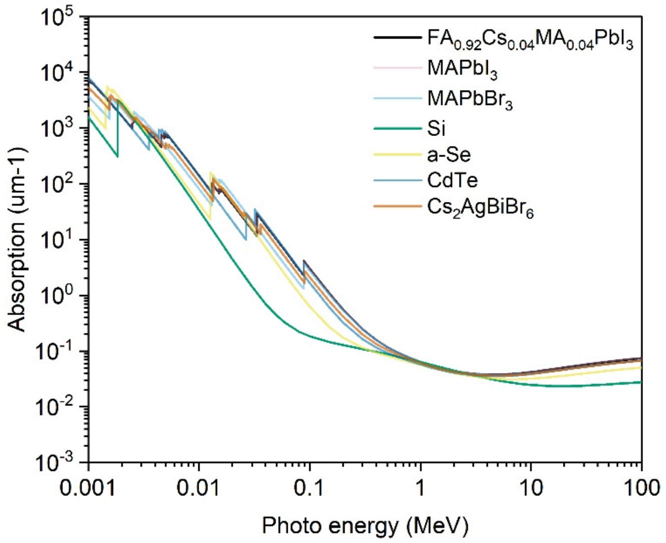

Figure S3. Absorption coefficient of $FA_{0.92}Cs_{0.04}MA_{0.04}PbI_3$ as a function of photon energy.

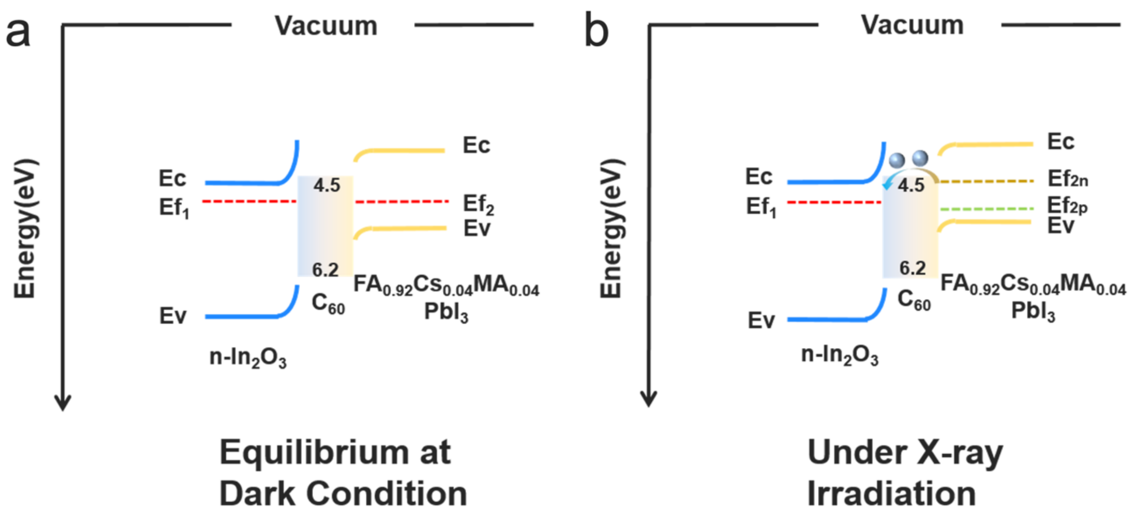

Figure S4. a, Band diagram of the device in the equilibrium state at dark. **b,** Band diagram of the device under X-ray irradiation.

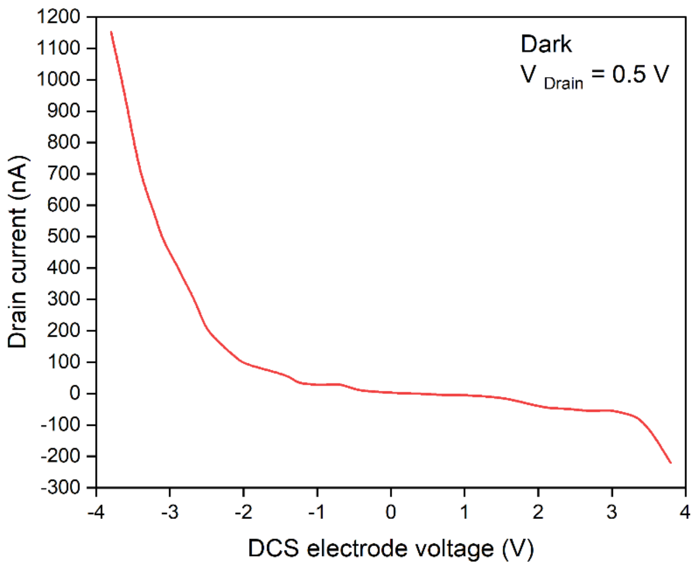

Figure S5. Current-voltage curves with respect to the DCS electrode voltage and the drain current. There is no gate regulation effect with this very thin PMMA spacing layer.

The sensitivity can be calculated by:

$$S = \frac{q}{D} = Q/AD't$$

where q is the charge density, D is the X-ray exposure dose at the detector, Q is the total charge induced by X-rays, A is the exposure area, D' is the dose rate of X-rays, and t is the X-ray exposure time.

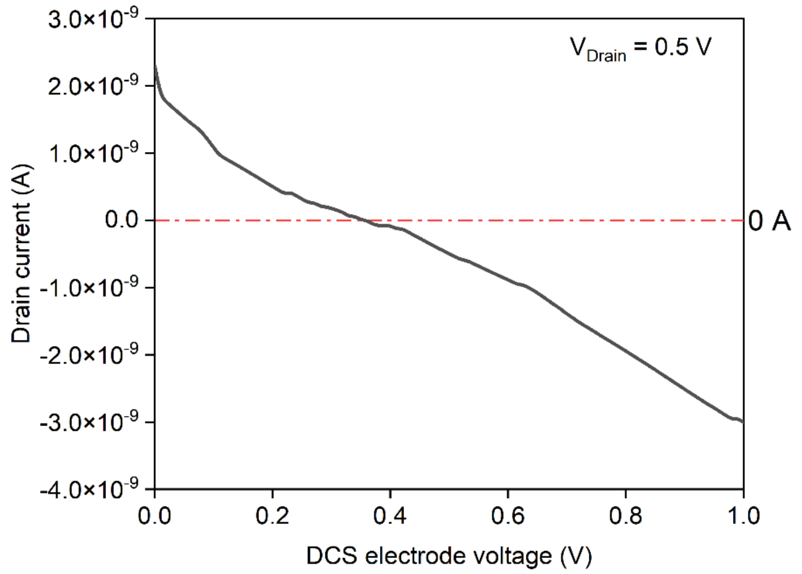

Figure S6. Current-voltage curves of the device without PMMA in terms of DCS electrode voltage and drain current at dark condition. The dark current can be diminished to zero while the DCS electrode is applied 0.4 V bias.

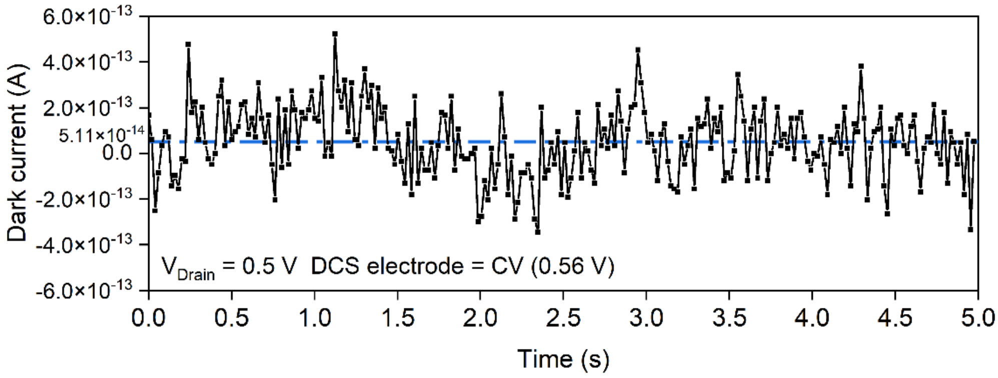

Figure S7. Truly measured dark current. The average dark current and noise current are 51.1 fA and 152 fA, respectively.

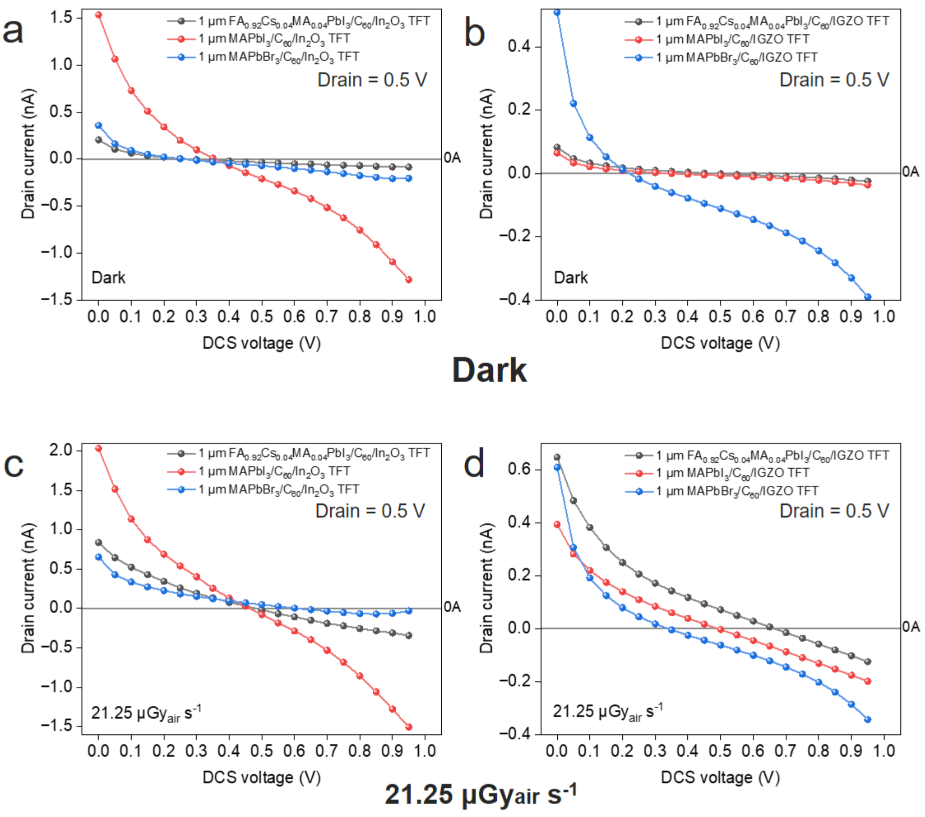

Figure S8. Current-voltage curves in terms of DCS electrode voltages and drain currents of devices with 1μm $MAPbBr_3$, $MAPbI_3$ and $FA_{0.92}Cs_{0.04}MA_{0.04}PbI_3$ on In_2O_3 and IGZO conduction channel in the dark and under X-ray. **a,** Current-voltage curves of 1μm $MAPbBr_3$, $MAPbI_3$ and $FA_{0.92}Cs_{0.04}MA_{0.04}PbI_3$ on C_{60}/In_2O_3 TFT in the dark. **b,** Current-voltage curves of 1μm $MAPbBr_3$, $MAPbI_3$ and $FA_{0.92}Cs_{0.04}MA_{0.04}PbI_3$ on C_{60}/IGZO TFT in the dark. **c,** Current-voltage curves of 1μm $MAPbBr_3$, $MAPbI_3$ and $FA_{0.92}Cs_{0.04}MA_{0.04}PbI_3$ on C_{60}/In_2O_3 TFT under 21.25 $\mu Gy_{air}\ s^{-1}$. **d,** Current-voltage curves of 1μm $MAPbBr_3$, $MAPbI_3$ and $FA_{0.92}Cs_{0.04}MA_{0.04}PbI_3$ on C_{60}/IGZO TFT under 21.25 $\mu Gy_{air}\ s^{-1}$.

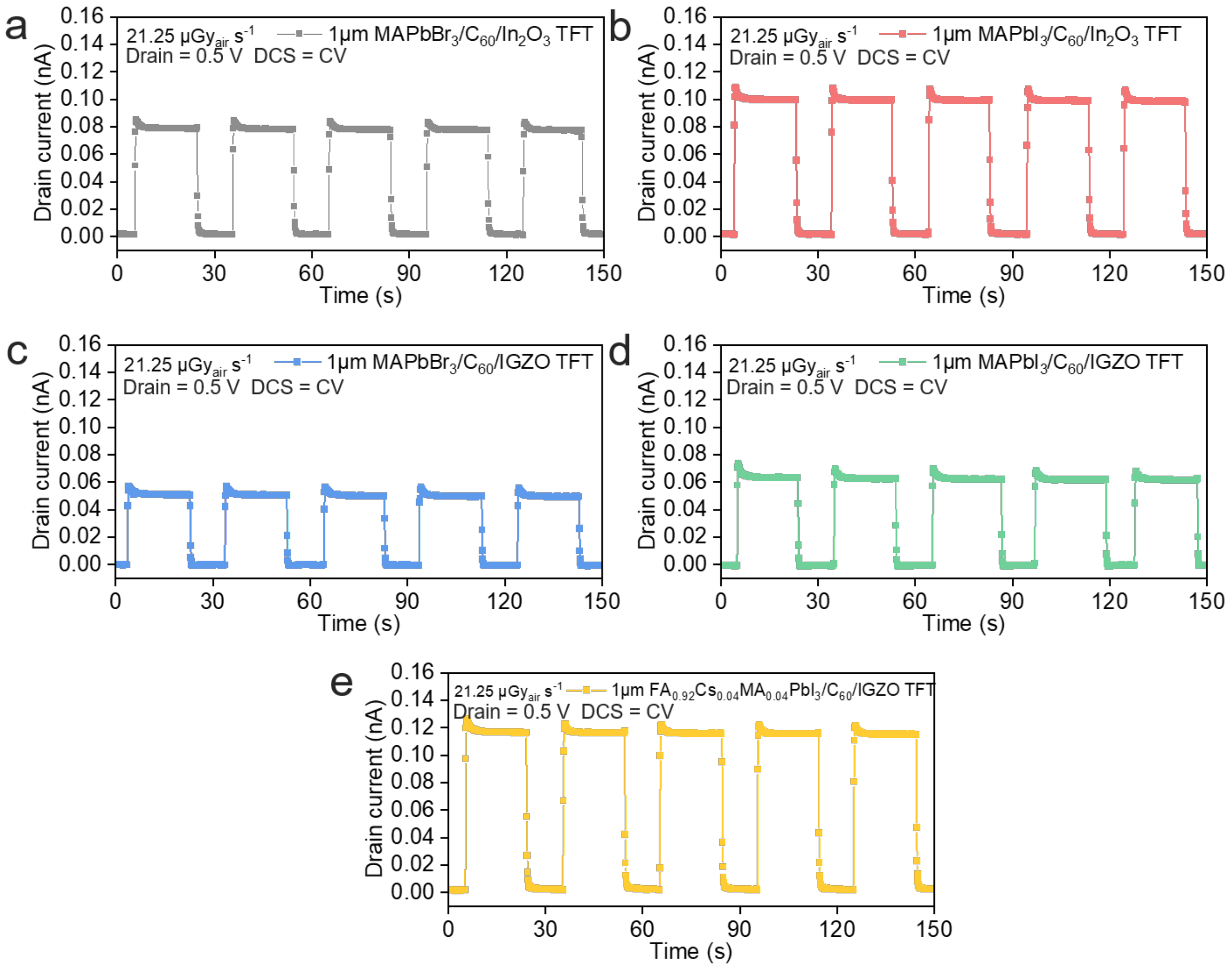

Figure S9. Pulse-train response of different perovskite and conduction channel materials based devices. The DCS electrode are all biased with CV. **a,** Pulse-train response of $MAPbBr_3/C_{60}/In_2O_3$ TFT under 21.25 μGy$_{air}$ s^{-1}. **b,** Pulse-train response of $MAPbI_3/C_{60}/In_2O_3$ TFT under 21.25 μGy$_{air}$ s^{-1}. **c,** Pulse-train response of $MAPbBr_3/C_{60}$/IGZO TFT under 21.25 μGy$_{air}$ s^{-1}. **d,** Pulse-train response of $MAPbI_3/C_{60}$/IGZO TFT under 21.25 μGy$_{air}$ s^{-1}. **e,** Pulse-train response of $FA_{0.92}Cs_{0.04}MA_{0.04}PbI_3/C_{60}$/IGZO TFT under 21.25 μGy$_{air}$ s^{-1}.

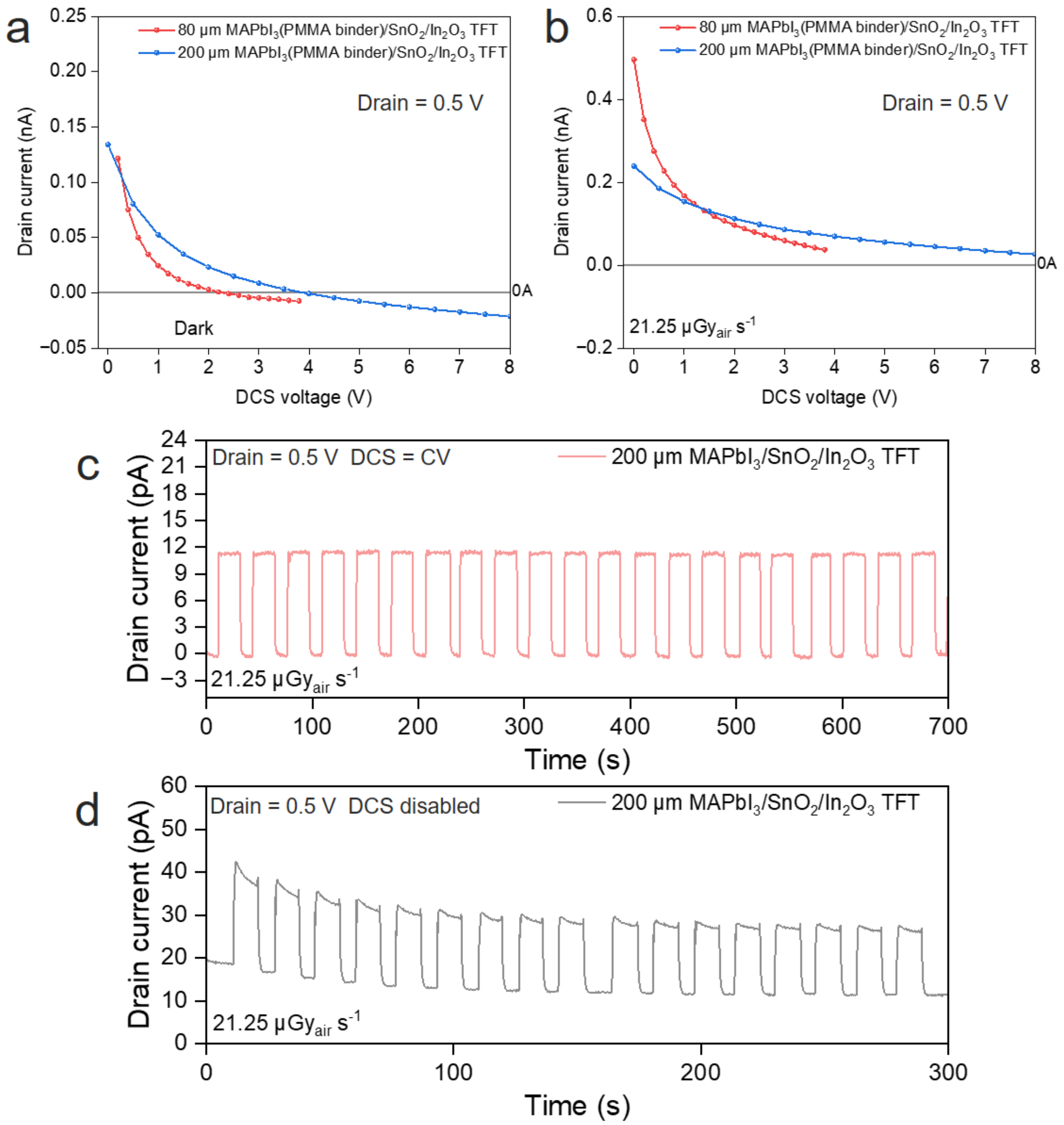

Figure S10. Performance of thick perovskite film devices. a, Current-voltage curves in terms of DCS electrode voltages and drain currents of 80 μm and 200 μm $MAPbI_3/SnO_2/In_2O_3$ TFT in the dark. **b,** Current-voltage curves in terms of DCS electrode voltages and drain currents of 80 μm and 200 μm $MAPbI_3/SnO_2/In_2O_3$ TFT under 21.25 μGy_{air} s^{-1}. **c,** Pulse-train response of 200 μm $MAPbI_3/SnO_2/In_2O_3$ TFT device under 21.25 μGy_{air} s^{-1}. The DCS electrode is biased with CV. d, Pulse-train response of 200 μm $MAPbI_3/SnO_2/In_2O_3$ TFT device under 21.25 μGy_{air} s^{-1}. The DCS electrode is disabled.

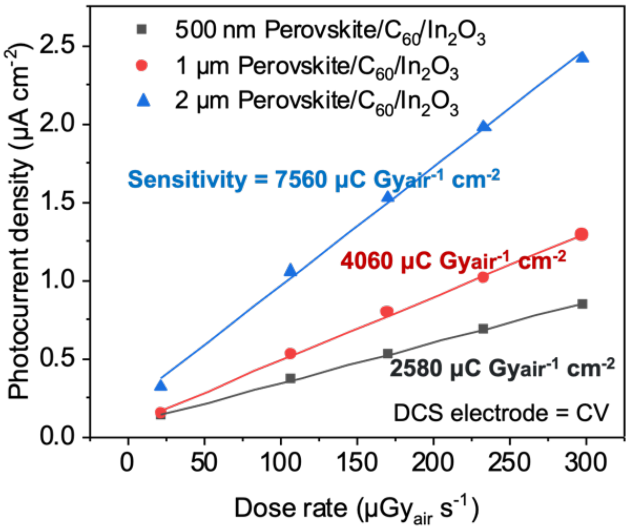

Figure S11. Sensitivity of 2 μm, 1 μm and 500 nm perovskite-based devices. The devices with thinner geometry have lower sensitivity.

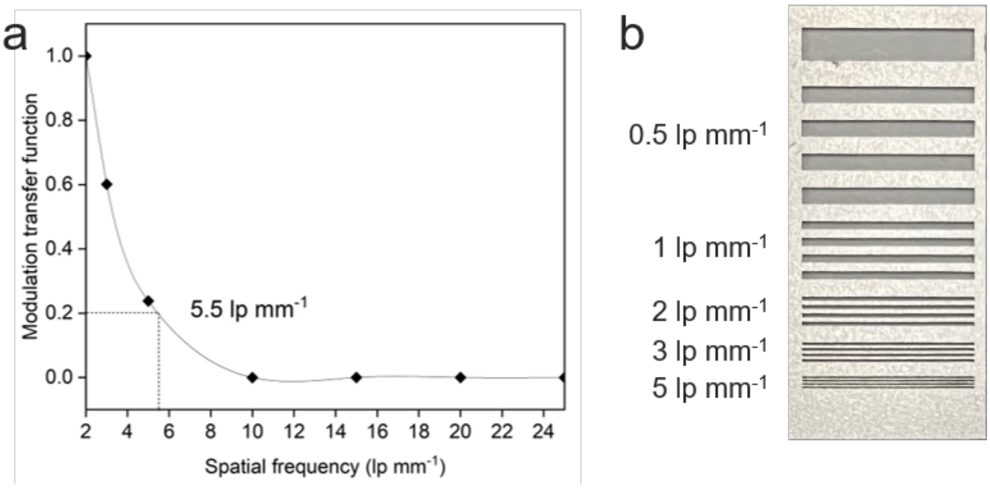

Figure S12. a, Modulation transfer function (MTF) of a single device. The minimum resolved line pair is 5.5 lp mm^{-1}. **b,** The used line pair mask plate

The MTF was calculated by:

$$\mathrm{MTF} = \frac{I_{max}-I_{min}}{I_{max}+I_{min}}$$

where I_{max} is the maximun current intensity and I_{min} is the minimum current intensity when detecting the line pairs.

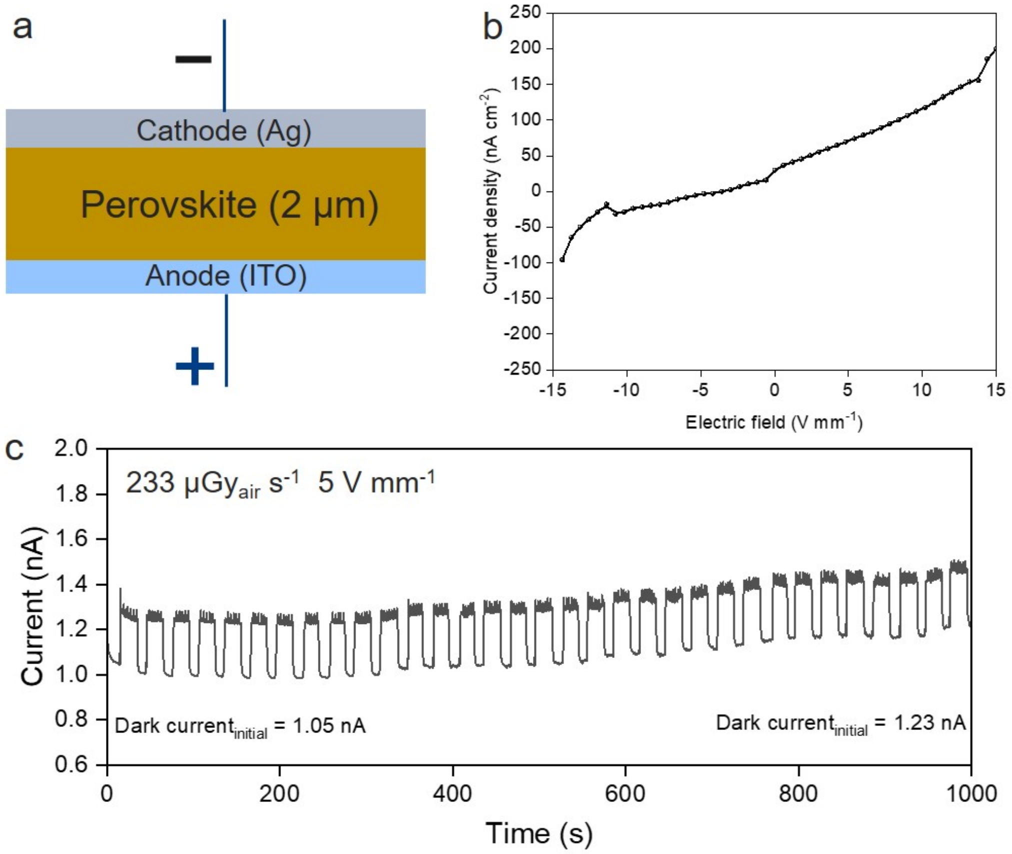

Figure S13. Optoelectronic properties of photoconduction detector. a, Illustration schematic of photoconductive (PCD) detector with a same material and a same thickness. **b,** Current-voltage curves of the PCD device. The dark current density is 72 nA cm^{-2} under the same electrical field with our DCS detector (5 V mm^{-1}). It is more than a thousand time higher than our DCS device. **c,** Pulse-trains of the PCD device under the exposure of the X-ray with a dose rate of 233 μGy_{air} s^{-1}. The dark current gradually shifts from 1.05 nA to 1.23 nA and the SNR is very poor.

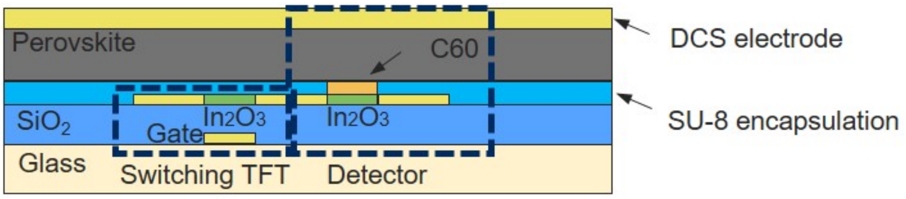

Figure S14. Sectional view of a single-pixel in the array. The DCS detector is in series with a switching TFT to control the on/off of a single-pixel.

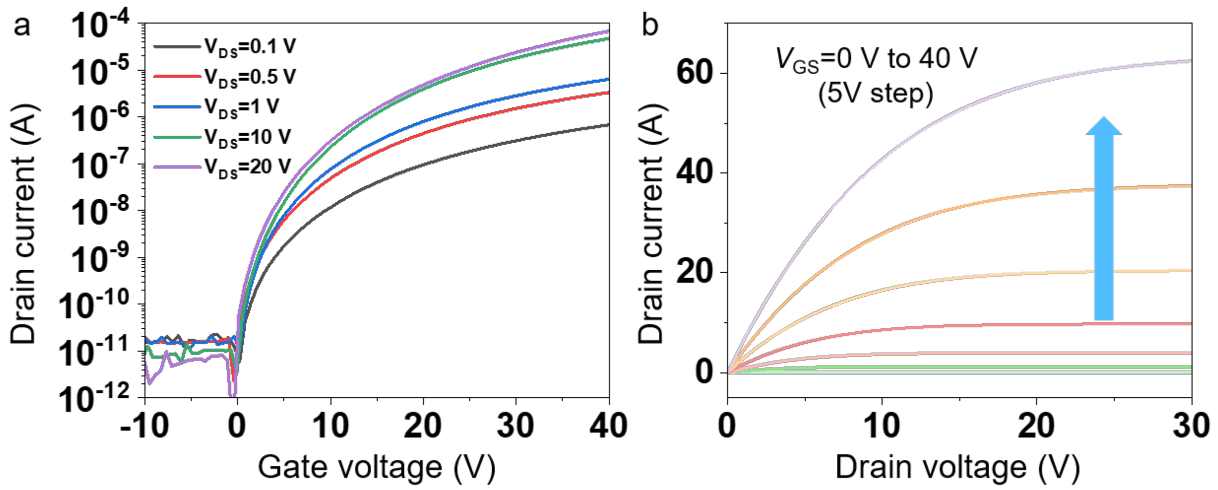

Figure S15. a, Transfer curves of a single transistor in the array. **b,** Output curves of a single transistor in the array.

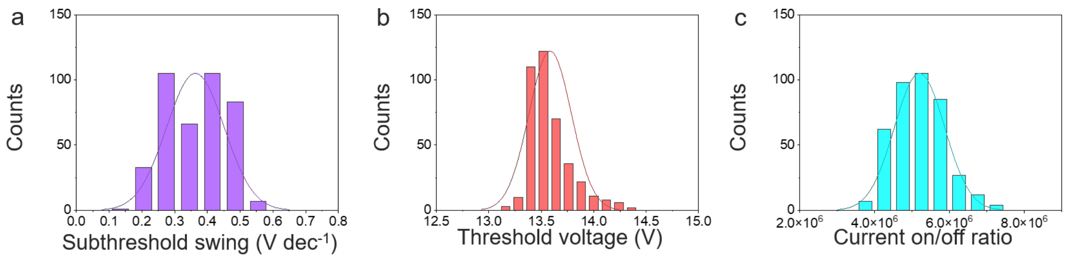

Figure S16. a, The subthreshold swing distribution of the 400 randomly selected pixels from the DCS detector array is demonstrated. **b,** The threshold voltage distribution of the 400 randomly selected pixels from the DCS detector array is demonstrated. **c,** The Current on/off ratio distribution of the 400 randomly selected pixels from the DCS detector array is demonstrated.

Where the linear mobility was calculated by:

$$\mu_{lin} = \frac{dI_D}{dV_{GS}} \times \frac{L}{W} \times \frac{1}{C_{ox} \times V_{DS}} \qquad (1)$$

where dI_D/dV_{GS} is the slope of the linear region in the transfer curve, L/W is the channel length-width ratio, C_{ox} is the gate insulator capacitance and V_{DS} is the drain electrode bias.

The S.S was calculated by:

$$\mathrm{S.S} = \frac{\partial V_{GS}}{\partial(\log_{10} I_D)} \qquad (2)$$

where V_{GS} is the gate electrode bias and I_D is the current of drain electrode.

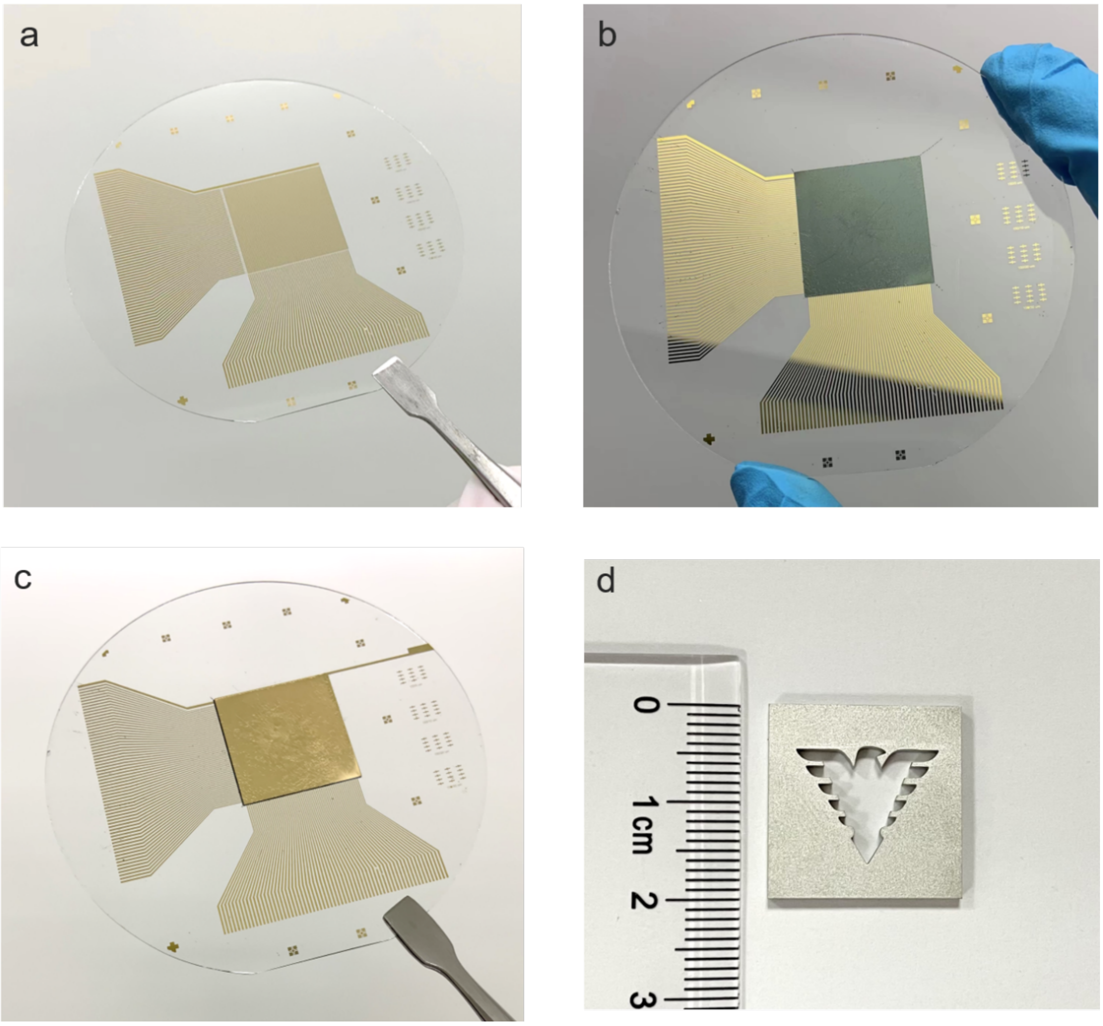

Figure S17. Intermediate process of DCS X-ray detectors array and mask plate. a, 64 x 64 matrix In_2O_3 TFTs back panel. Its pixel density is as high as 1024 per square centimeter. **b,** A 50 nm layer of C_{60} was first evaporated on the back panel and then a 2 um layer of perovskite was spin-coated above the area of the detector array. **c,** With a layer of DCS electrode evaporated onto the area of the detector array, the fabrication of the DCS detector array completed. **d,** Image of the 2 mm thick stainless steel mask plate with a hollowed-out figure of 'Qiushi Eagle'.

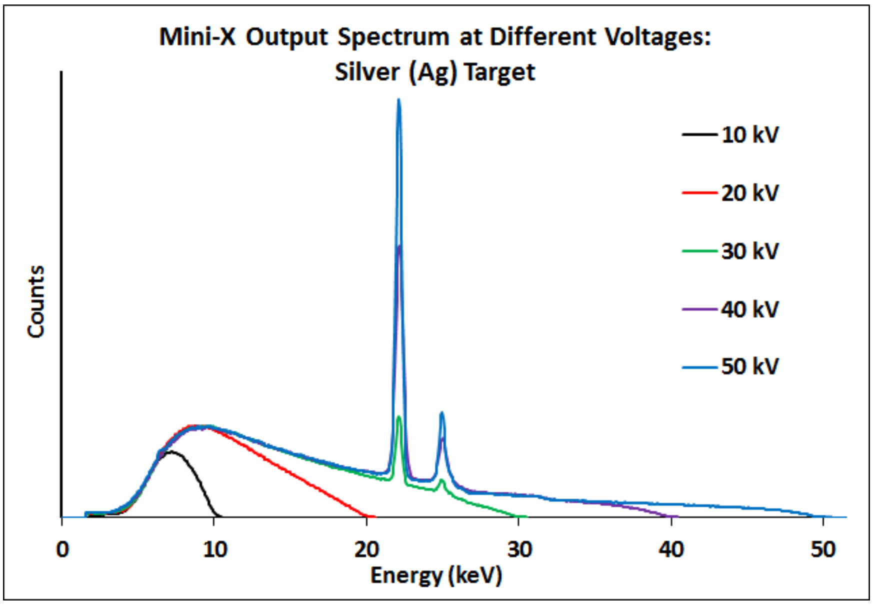

Figure S18. The used X-ray output spectrum at different voltages.
